# Effects of Mind–Muscle Connection on Muscle Activity During Machine-Based Shoulder Press in Untrained Individuals

**DOI:** 10.3390/jcm15103925

**Published:** 2026-05-20

**Authors:** Donghee Kim, Jonggeun Woo, Seungryeol Lee, Jungu Jung, Dongyeop Lee, Jiheon Hong, Jaeho Yu, Jinseop Kim, Yeongyo Nam, Jeongwoo Jeon

**Affiliations:** 1 Department of Physical Therapy, College of Health Sciences, Sunmoon University, Asan-si 31460, Republic of Korea; pjk00006@naver.com (D.K.); jgw9968@naver.com (J.W.); atctk0906@naver.com (S.L.); jms58202@naver.com (J.J.); leedy@sunmoon.ac.kr (D.L.); hgh1020@hanmail.net (J.H.); naresa@sunmoon.ac.kr (J.Y.); skylove3373@sunmoon.ac.kr (J.K.); nyg3583@hanmail.net (Y.N.); 2 Digital Healthcare Institute, Sunmoon University, Asan-si 31460, Republic of Korea

**Keywords:** mind–muscle connection, electromyography, muscle activity, resistance training, machine-based shoulder press

## Abstract

**Background/Objectives**: Mind–muscle connection (MMC) refers to a strategy in which an individual intentionally focuses attention on specific muscles to enhance the neural activation of those muscles. The purpose of this study was to examine the acute effects of MMC directed toward a specific muscle during a machine-based shoulder press exercise on the muscle activity of untrained individuals. **Methods**: Thirty-one healthy young adults with no athletic or resistance training experience participated in this single-session study. Participants performed machine-based shoulder presses at 40% of one-repetition maximum intensity under three MMC conditions. The three conditions were: no-focus condition, deltoid (DT)-focused condition, and triceps brachii (TB)-focused condition. Muscle activities of the DT, TB, and upper trapezius (UT) were measured during exercise under each MMC condition. Differences in muscle activity across conditions were analyzed using a two-way repeated-measures analysis of variance. **Results**: A total of 31 participants were included in the final analysis. A significant interaction between muscle and MMC condition was observed (*p* < 0.001). Post hoc analysis showed that DT activity was higher in the DT-focused condition, whereas TB activity was higher in the TB-focused condition (*p* < 0.001). UT activity did not differ across conditions (*p* > 0.05). **Conclusions**: MMC selectively enhanced target muscle activity without changing non-target muscle activity, indicating its potential for selective neuromuscular recruitment. These findings suggest that MMC may serve as a practical strategy for selective muscle activation during machine-based resistance training. Further study is needed to determine whether these findings translate into long-term improvements in muscle strength and functional performance.

## 1. Introduction

Resistance training involves the generation of muscular force against external resistance and is widely used to enhance muscular strength, hypertrophy, and endurance [1,2]. Regular resistance training is associated with multiple health benefits, including improved muscle function, joint stability, bone mineral density, and reduced risk of cardiovascular and metabolic diseases [3,4]. However, compared with well-trained individuals, untrained individuals often exhibit less-efficient movement strategies during resistance training, which may limit their ability to activate the target muscles [5,6,7]. Consequently, excessive co-activity of synergistic or stabilizing muscles may occur, limiting the selective activity of specific muscles [8]. Accordingly, machine-based exercises that provide a stable movement trajectory and reduce the activity demand of stabilizing muscles may be advantageous compared with free-weight exercises, which allow greater degrees of freedom at the joints [9,10,11].

In addition to these exercise-related factors, sensory cues such as verbal instructions, tactile feedback, and imagery training are frequently used to enhance the selective activity of target muscles during resistance exercise [12]. Recently, the mind–muscle connection (MMC) has received increasing attention as a strategy in which individuals consciously direct attention to a specific muscle to facilitate neural drive [13,14]. Indeed, MMC has been proposed to increase electromyographic (EMG) activity of the target muscle, which has been suggested to contribute to improvements in muscle strength and hypertrophy in trained individuals [14,15]. Furthermore, cognitive attentional focus alone has been reported to modulate cortical activity and may be accompanied by increased neuromuscular activity [16,17,18]. These findings indicate that MMC may induce selective muscle activity without altering the external load [19]. Accordingly, integrating MMC into resistance training may represent a promising approach to enhancing selective muscle activation during exercise.

During resistance exercise, focusing attention on the agonist muscle may alter the activity patterns of both the agonist and synergistic muscles, potentially influencing the overall movement strategy [20,21]. However, it remains unclear whether the increased target muscle activity during MMC is accompanied by increased activity of synergistic muscles or occurs at the expense of activity in other muscles [14,15]. Therefore, the purpose of this study was to examine the effects of MMC directed toward a specific muscle during a machine-based shoulder press exercise on the muscle activity of the deltoid (DT), triceps brachii (TB), and upper trapezius (UT) in untrained individuals. Specifically, this study aimed to determine whether MMC selectively increases activity of the target muscle or is accompanied by changes in the activity patterns of other muscles. We hypothesized that MMC would selectively increase the activity of the target muscle without changing the activity of other muscles.

## 2. Materials and Methods

### 2.1. Study Design

This study employed a randomized repeated-measures design. The trial was conducted at the Department of Physical Therapy, Sunmoon University. The study protocol was approved by the Institutional Review Board of Sunmoon University (IRB No. SM-202511-026-2). All procedures were conducted in accordance with the ethical principles of the Declaration of Helsinki. All participants provided written informed consent prior to participation.

### 2.2. Sample Size

Sample size calculation was performed using a statistical software (G-Power 3.1.9.7, Düsseldorf, Germany) for a two-way repeated-measures analysis of variance (ANOVA). Previous studies have reported large interaction effects of MMC-related interventions on maximal workload [13], whereas studies examining EMG responses have shown small-to-moderate effects [20]. Therefore, considering the differences in outcome measures and study designs, a conservative moderate effect size (f = 0.25) was assumed for the sample size calculation. With a significance level (α) of 0.05 and a statistical power (1 − β) of 0.80, the minimum required sample size was 28 participants. Accounting for an anticipated dropout rate of approximately 10%, the target sample size was set at 31 participants.

### 2.3. Participants

Participants were recruited through online and offline advertisements within the university and community. The inclusion criteria were based on a previous study defining untrained individuals and included healthy young adults who had no history of athletic training or structured resistance training experience [22]. All participants were instructed not to engage in any other exercise programs during the study period. The exclusion criteria were as follows: (1) current or chronic shoulder pain; (2) a history of shoulder surgery or a diagnosis of rotator cuff injury; (3) shoulder pain lasting more than two consecutive weeks within the previous year; (4) a history of upper-extremity surgery or injury; and (5) neurological or cardiovascular conditions that could limit participation in the study [23].

### 2.4. Experimental Procedures

Participants were required to visit the laboratory on three occasions, each separated by a 24-h interval. Caffeine and analgesic intakes were restricted for at least 12 h before each visit. All measurements were conducted at the same time of day. The three visits consisted of a warm-up, measurement of maximal voluntary isometric contraction (MVIC) of the DT, TB, and UT muscles, and measurement of muscle activity during machine-based shoulder press, respectively. For the dynamic warm-up, each participant performed a 10-min ergometer cycling session at a self-selected pace to achieve submaximal intensity. At the first visit, participants’ demographic characteristics, including age, height, weight, and dominant hand, were recorded. All participants completed the shoulder press exercise in a no-focus condition on the first day. During the second and third visits, participants performed the shoulder press exercise under one MMC condition per visit (DT-focused or TB-focused), with the order randomized. Participants were not informed of the specific hypotheses of the study or the expected outcomes of each condition.

### 2.5. Machine-Based Shoulder Press

**Figure 1:**
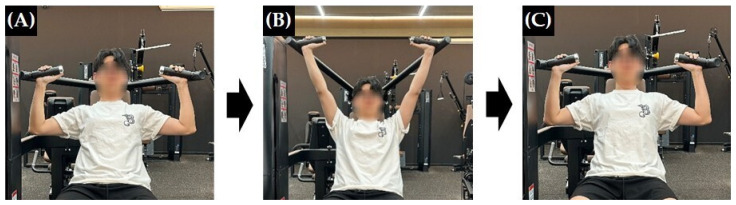
Machine-based shoulder press exercise. (**A**) Starting position. (**B**) Pressed position. (**C**) Return position.

The shoulder press exercise was performed using a resistance training machine (LS-701 multi-press machine, LEXCO, Daegu, Republic of Korea). In the starting position, participants were seated with the backrest set at 75°, maintaining trunk contact with the backrest. The feet were positioned shoulder-width apart with the knees flexed at approximately 90°. The elbows were flexed at approximately 90°, and the grip position was kept consistent across all conditions. Following the researcher’s verbal instructions, participants pushed the handles upward until the elbows were fully extended. The handles were then lowered back to the level of the shoulders (Figure 1). Participants performed the exercise at a self-selected pace. During the exercise, participants were monitored by the researcher to ensure that the elbows moved parallel to the frontal plane and that no compensatory trunk movements occurred [24]. Exercise intensity was set at 40% of the one-repetition maximum (1RM), which was estimated using the Brzycki equation (1RM = weight/(1.0278 − 0.0278 × repetition)). The 40% 1RM load was considered a moderate intensity based on a previous study [15]. A moderate intensity was selected because higher intensities may shift attentional focus toward task completion rather than internal focus on the target muscle. Participants completed three sets of 10 repetitions under each condition, with a rest period of at least 3 min between sets to minimize fatigue [24,25].

### 2.6. Mind–Muscle Connection

Three attention conditions were applied for the MMC during machine-based shoulder presses: (1) a control condition with no attention directed toward specific muscles (no-focus condition); (2) an internal focus condition targeting the DT (DT-focused condition); and (3) an internal MMC condition targeting the TB (TB-focused condition). In the no-focus condition, participants completed the exercise without any instructions. The MMC conditions required participants to focus their attention on the contraction of target muscles (DT or TB) during shoulder presses, reflecting an internal focus strategy rather than an external focus strategy [15]. Prior to exercise, information on the anatomical location of each muscle was provided to ensure accurate recognition of the target muscle Additionally, the same researcher provided all participants with standardized verbal instructions and light tactile cues to enhance awareness of the target muscle in the MMC condition. To sustain attentional focus, participants were instructed before each set to “focus on the contraction of the DT or TB”.

### 2.7. Muscle Activity

**Figure 2:**
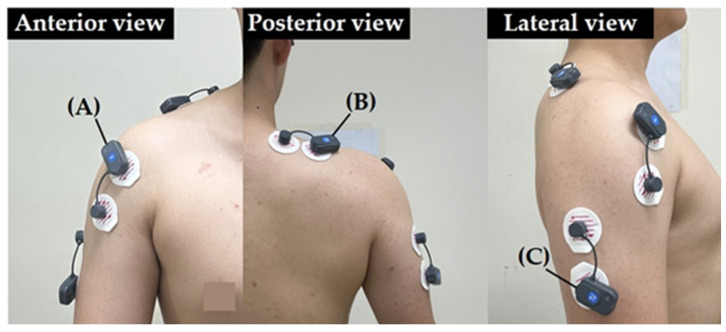
Surface electromyography electrode placement. (**A**) Anterior deltoid; (**B**) Upper trapezius (**C**) Triceps brachii lateral head.

Muscle activity of the dominant DT, TB, and UT during machine-based shoulder press exercise was recorded using a wireless surface EMG system (FREE EMG 1000, BTS Bioengineering, Milan, Italy). EMG signals were sampled at 1000 Hz, band-pass filtered (20–450 Hz) using a fourth-order Butterworth filter, full-wave rectified, and smoothed using a root mean-square (RMS) with a 100-ms moving window. Before electrode placement, the skin over each muscle was cleaned with an alcohol swab to reduce impedance. Electrode placement was performed by the same trained investigator for all participants to ensure consistency. The placement of bipolar Ag/AgCl surface electrodes (diameter: 10 mm; inter-electrode distance: 20 mm) followed the SENIAM (Surface Electromyography for the Non-Invasive Assessment of Muscles) guidelines (Table 1, Figure 2). The electrodes were placed parallel to the direction of the muscle fibers.

Maximal voluntary isometric contractions (MVIC) for each muscle were measured before the shoulder press exercise. Prior to MVIC testing, participants completed two submaximal practice trials to ensure familiarity with the testing procedure. For the DT, MVIC was assessed with the shoulder abducted to 90° with external rotation and the elbow flexed to 110°, with resistance applied via a machine fixed at the shoulder press angle [24]. For the TB lateral head, participants were seated with the shoulder flexed to 90° and the elbow flexed to 90° in a neutral forearm position, with the distal humerus stabilized and resistance applied against elbow extension [26]. For the UT, MVIC was measured with the shoulder abducted to 90°, the neck side-bent to the same side, rotated to the opposite side, and extended, with resistance applied simultaneously to shoulder abduction and the head [27]. The MVIC for each muscle was performed for 5 s across three trials, with a 2-min rest interval between trials to minimize fatigue. The mean RMS value of the middle 3 s from each trial was calculated, and the highest value among the three trials was used for normalization.

EMG signals recorded during the shoulder press exercise were analyzed over the full repetition cycle, including both concentric and eccentric phases. The onset and offset of each repetition were visually identified from the EMG signals by a skilled investigator based on changes in signal amplitude corresponding to each repetition [28]. This process was completed by the same investigator for all participants to ensure consistency. To ensure reliability, another investigator independently reviewed a subset of the data, and any discrepancies were resolved by consensus. The RMS value was calculated for each repetition, and the mean RMS value across repetitions within each condition was used for analysis. EMG amplitude was normalized to MVIC and expressed as a percentage of MVIC (%MVIC).

### 2.8. Statistical Analysis

All statistical analyses were performed using IBM SPSS Statistics (version 26.0, IBM Corp., Armonk, NY, USA). All data are presented as mean ± standard deviation. Normality was assessed using the Shapiro–Wilk test, and homogeneity of variance was evaluated using Levene’s test. Differences in muscle activity across conditions were analyzed using a two-way repeated-measures ANOVA, with muscle (DT, TB, and UT) and MMC condition (no-focus, DT-focused, and TB-focused). When significant main effects or interactions were found, post hoc pairwise comparisons with Bonferroni correction were performed to examine differences between conditions within each muscle and between muscles within each condition. A two-tailed *p*  <  0.05 was considered statistically significant. Effect sizes were expressed as partial eta squared (η_p_^2^), with values of 0.01, 0.06, and 0.14 interpreted as small, medium, and large, respectively.

## 3. Results

A total of 31 participants (15 males and 16 females) were enrolled in this study, and all were included in the final analysis. The general characteristics of the participants are presented in Table 2.

A two-way repeated-measures ANOVA revealed a significant muscle × MMC condition interaction (F (4, 120) = 77.55, *p* < 0.001), as well as significant main effects of muscle (F (2, 60) = 14.42, *p* < 0.001) and MMC condition (F (2, 60) = 34.62, *p* < 0.001) (Table 3).

**Figure 3:**
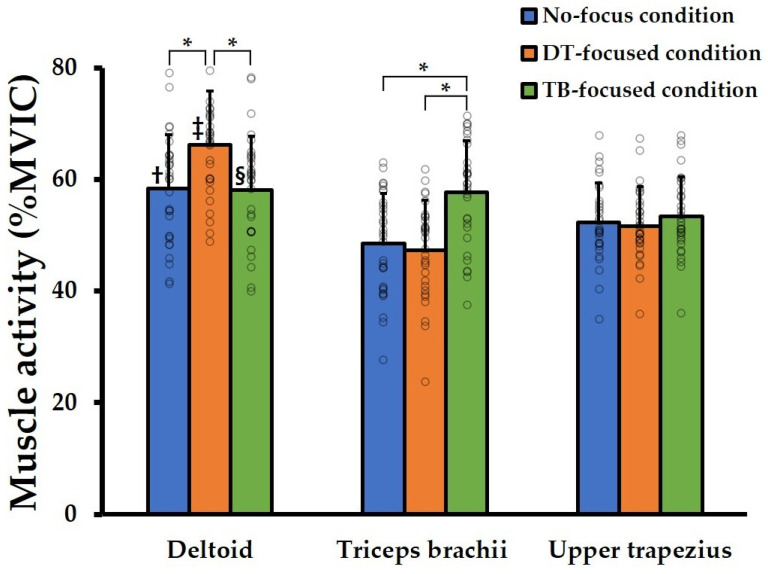
Muscle activity of the deltoid, triceps brachii, and upper trapezius during machine-based shoulder press exercise under mind–muscle connection conditions. * Significant differences in muscle activity between conditions (*p* < 0.001). ^†,‡^ DT activity was higher than that of TB and UT activities in both the no-focus (*p* < 0.01) and DT-focused (*p* < 0.001) conditions. ^§^ In the TB-focused condition, DT activity was higher than that of UT (*p* < 0.05). MVIC, maximal voluntary isometric contraction; DT, deltoid; TB, triceps brachii; UT, upper trapezius.

The results of the post hoc analysis are presented in Figure 3. Pairwise post hoc comparisons are reported as mean difference (MD) with 95% confidence interval (CI) and *p*-values. DT muscle activity was significantly greater in the DT-focused condition than in the no-focus (MD = 7.86, 95% CI = 5.70–10.02, *p* < 0.001) and TB-focused conditions (MD = 8.19, 95% CI = 6.05–10.34, *p* < 0.001). TB activity was significantly higher in the TB-focused condition compared with the no-focus (MD = 9.16, 95% CI = 7.12–11.21, *p* < 0.001) and DT-focused conditions (MD = 10.37, 95% CI = 8.22–12.52, *p* < 0.001). No significant differences in UT activity were observed among the three conditions (*p* > 0.05).

Comparisons of muscle activity within MMC conditions indicated that DT activity was higher than that of TB and UT activities in both the no-focus (DT vs. TB: MD = 9.91, 95% CI = 3.99–15.83, *p* = 0.001; DT vs. UT: MD = 6.05, 95% CI = 1.46–10.63, *p* = 0.007) and DT-focused (DT vs. TB: MD = 18.98, 95% CI = 13.02–24.94, *p* < 0.001; DT vs. UT: MD = 14.64, 95% CI = 10.43–18.85, *p* < 0.001) conditions. In the TB-focused condition, DT activity was higher than that of UT (MD = 4.70, 95% CI = 0.38–9.02, *p* = 0.029).

## 4. Discussion

This study investigated the effects of MMC, an internal focus on specific muscles, on muscle activity levels during machine-based shoulder press exercise. Previous studies examining the effects of MMC on selective muscle activity have primarily been conducted in individuals with resistance training experience. Accordingly, the present study investigated the effects of MMC under relatively stable exercise conditions in untrained individuals. The primary finding was that focusing attention on specific muscles during machine-based shoulder press exercise increased muscle activity. Furthermore, there was no accompanying increase in the activity of non-target muscles. These results suggest that MMC may promote selective neuromuscular activity of target muscles under the machine-based shoulder press condition used in this study.

Changes in attention focus through MMC during resistance training may have induced increased muscle activity by regulating muscle recruitment strategies during exercise performance. A recent study demonstrated that attentional focus induces differences in corticospinal excitability and intracortical regulation during dynamic exercise [29]. Accordingly, the authors suggested that attentional focus can influence the central nervous system. Thus, internal focus on the target muscle may have induced muscle activity through modulation of neural regulation. In relation to resistance training, it has been shown that focusing on specific muscles while performing bench presses increases muscle activity in resistance-trained individuals [15]. Similarly, MMC induced through verbal instruction during the bench press was shown to increase TB activity in resistance-trained males [20]. These studies have mainly been conducted in resistance-trained individuals; however, some evidence indicates that changes in muscle activity associated with an internal focus are not necessarily dependent on prior resistance training experience [30]. In the present study, increased awareness of the DT and TB through verbal instructions and tactile cues (internal focus) during a machine-based shoulder press led to increased muscle activity in untrained individuals. These findings suggest that MMC may serve as a potential strategy for promoting selective neuromuscular activity during machine-based resistance exercise in untrained individuals.

During the machine-based shoulder press, activity of the DT and TB varied according to the attentional MMC condition, whereas UT activity did not differ across conditions. These findings indicate that MMC facilitated the selective activity of the target muscles rather than a generalized increase in muscle recruitment. Untrained individuals tend to exhibit increased coactivity of antagonist and synergist muscles as a compensatory strategy to enhance joint stability when performing motor tasks [31,32,33]. For instance, during the free-weight bench press, an internal focus on a specific muscle has been associated with increased activity in both target and synergistic or alternate muscles [20]. However, the absence of changes in the synergist muscle (UT) activity across the three conditions (no-focus, DT-focused, and TB-focused) in the present study indicates that MMC did not elicit such compensatory co-contraction. This discrepancy may be explained by differences in mechanical demands between exercise modalities. Free-weight exercises involve greater degrees of freedom and require increased stabilization, which may lead to more generalized increases in muscle activity [34,35]. In contrast, the machine-based exercise used in this study provided a mechanically constrained environment that reduced postural control demands, thereby allowing more focused recruitment of the target muscles without additional activity of surrounding muscles. Moreover, the present study employed a low-load corresponding to 40% of 1RM during resistance exercise. Considering that in high-load conditions, individuals may shift their attentional focus toward task execution (i.e., lifting the load) rather than specific muscles, a low-load may have been more conducive to eliciting selective activity through MMC during resistance exercise [15]. Therefore, the increased activity of the target muscle observed in the present study seems to reflect selective neural recruitment under mechanically stable conditions. Taken together, these findings indicate that MMC may enhance selective activity of target muscles without increasing compensatory activity of synergistic muscles when movement is mechanically constrained.

This study has several limitations that should be considered. The present findings are limited to the immediate neuromuscular responses to MMC observed within a single-session, repeated-measures experimental design, and therefore do not provide insight into the potential long-term effects. In our experimental protocol, the first session was fixed as the no-focus condition, while the second and third sessions were randomly assigned to either the DT- or TB-focused conditions. This approach was adopted because performing MMC conditions first may influence subsequent sessions through short-term motor learning or altered neuromuscular recruitment associated with focusing on a specific muscle. Nevertheless, potential order effects cannot be completely excluded in this study. In addition, the relatively low exercise intensity (40% 1RM) may limit the generalizability of these findings to higher-intensity resistance training conditions. Furthermore, these findings are specific to the machine-based shoulder press exercise in untrained individuals and should be interpreted with caution when applied to other exercises, modalities, or populations. Finally, surface EMG amplitude is an indirect marker of muscle activation and should not be equated with direct measures of force production or functional performance. Thus, the findings should be interpreted as evidence of altered neuromuscular recruitment patterns rather than direct functional improvement.

## 5. Conclusions

This study investigated the effects of targeted internal attention, referred to as the MMC, on muscle activity during a machine-based shoulder press exercise in untrained individuals. The results demonstrated that MMC selectively increased the activity of target muscles without increasing the activity of non-target muscles. Specifically, the DT and TB showed significant increases in muscle activity when attention was focused on each respective muscle, whereas UT activity did not differ across conditions. These findings suggest that MMC may influence selective neuromuscular recruitment patterns in untrained individuals under relatively low-intensity (40% 1RM) and mechanically constrained resistance exercise conditions, such as machine-based shoulder press. Further studies should examine the effects of MMC across a broader range of exercise modalities, intensities, and populations. In addition, it remains to be determined whether the acute increases in target muscle activity observed with MMC translate into long-term training adaptations.

## Figures and Tables

**Table 1:** Placement of surface electromyography electrodes.

Muscle	Placement
Anterior deltoid	Located anterior and distal to the acromion (~1 finger breadth).
Triceps brachii (Lateral head)	Placed at 50% of the line between the posterior acromial crest and the olecranon, positioned laterally (~2 finger breadths) from the line.
Upper trapezius	Located at 50% of the line between the acromion and the spinous process of C7.

**Table 2:** General characteristics of the participants.

Variables	Total (*n* = 31)	Male (*n* = 15)	Female (*n* = 16)
Age (years)	24.71 ± 2.27	25.47 ± 2.56	24.00 ± 1.75
Height (cm)	169.32 ± 6.82	175.00 ± 3.80	164.00 ± 4.15
Weight (kg)	66.00 ± 9.27	73.20 ± 6.95	59.25 ± 5.12
Shoulder press 1RM (kg)	29.19 ± 14.67	42.67 ± 7.76	16.56 ± 4.73

Values are presented as mean ± standard deviation. 1RM, one repetition maximum.

**Table 3:** Muscle activity of the deltoid, triceps brachii, and upper trapezius during machine-based shoulder press exercise under the mind–muscle connection conditions.

Muscle Activity (%MVIC)	MMC Conditions			*p*-Value (η_p_^2^)		
	No-Focus	DT-Focused	TB-Focused	Muscle	MMC	Muscle by MMC
DT	58.42 ± 9.63	66.28 ± 9.04	58.09 ± 9.68	<0.001 (0.325)	<0.001 (0.536)	<0.001 (0.721)
TB	48.51 ± 9.03	47.30 ± 8.49	57.67 ± 9.34			
UT	52.37 ± 7.07	51.64 ± 6.36	53.39 ± 7.14			

Values are presented as mean ± standard deviation. MVIC, maximal voluntary isometric contraction; MMC, mind–muscle connection; DT, deltoid; TB, triceps brachii; UT, upper trapezius.

## Data Availability

The data presented in this study are available on request from the corresponding author.

## Abbreviations

The following abbreviations are used in this manuscript:

DT	Deltoid
CI	Confidence interval
EMG	Electromyography
MD	Mean difference
MMC	Mind–muscle connection
MVIC	Maximal voluntary isometric contraction
RMS	Root mean square
TB	Triceps brachii
UT	Upper trapezius
1RM	One-repetition maximum
